# A Practical Approach to Platelet Phenotype Profiling Using Microplate Aggregometry

**DOI:** 10.3390/ph19060821

**Published:** 2026-05-23

**Authors:** Cezary Watala, Jacek Golański, Magdalena Boncler

**Affiliations:** Department of Haemostasis and Haemostatic Disorders, Medical University of Lodz, Mazowiecka 6/8, 92-215 Lodz, Polandjacek.golanski@umed.lodz.pl (J.G.)

**Keywords:** blood platelets, platelet phenotyping, platelet pharmacology, aggregation, stratification analysis

## Abstract

**Background/Objectives**: Blood platelets exhibit substantial functional heterogeneity, yet no established principles exist for distinguishing their subpopulations. The present study proposes a methodology for the evaluation of platelet reactivity and inhibitor sensitivity, with the aim of facilitating the expeditious identification of platelet phenotypes under standard laboratory conditions. **Methods**: The phenotyping of healthy subjects was based on the study of platelet aggregation in response to agonists and inhibitors of P2Y_12_, PAR-1 and GPVI receptors. The classification of variants was conducted on the basis of the similarities and differences in EC_50_/IC_50_ values obtained for individual ligands. Subsequently, the values were subjected to two- and six-variable cluster analyses. **Results**: Two major clusters (variants) were identified with consistent reliability across the range of analytical strategies employed. Cluster 1 comprised individuals with low EC_50_ values and moderate to high IC_50_ values, indicating high agonist responsiveness and relatively low inhibitor sensitivity. Conversely, cluster 2 exhibited the inverse pattern, characterised by moderate to high EC_50_ values and moderate to low IC_50_ values. Cluster 1 constituted a significant proportion of individuals (29–78%, depending on the analysis). The study did not identify a “low responder group”. **Conclusions**: The proposed methodology is distinguished by two features: its flexibility and its accessibility. These characteristics enable the identification of any platelet phenotype associated with selected signalling pathway(s). The application of this approach has the potential to facilitate the identification of individuals at elevated cardiovascular risk, thereby informing personalised antiplatelet therapy in the context of primary prevention.

## 1. Introduction

Blood platelets play a fundamental role in physiological haemostasis, but are also involved in the development of the inflammatory response and thrombosis [1]. Experimental observations accumulated over decades indicate that platelets are structurally and functionally heterogeneous. Even subtle changes in platelet size, morphology, activation, reactivity, and other properties influence their phenotypic and functional fate in physiological contexts. This diversity allows for the classification of platelet subpopulations into distinct reactive groups across both healthy individuals and patients [2,3]. As Thomas et al. [4] have highlighted, such diversity in platelets arises at multiple levels. These range from population-wide influences, such as age, sex, and race, to variations within individuals driven by megakaryocyte heterogeneity, platelet ageing, and environmental cues encountered in circulation. For example, studies have shown that African Americans may exhibit lower platelet reactivity, both at baseline and during antiplatelet therapy than European Americans, demonstrating race-related differences in platelet response [5]. Furthermore, large-scale transcriptomic, proteomic and lipidomic studies further demonstrate that disease states, genetic factors, and therapeutic interventions further refine these distinct phenotypes. Taken together, this growing body of evidence highlights the importance of accounting for platelet heterogeneity when interpreting function and designing precision diagnostic or therapeutic strategies in research and clinical practice [4].

There are numerous approaches, involving different variables and methods, to investigate the diverse aspects of platelet function. Importantly, complementary or alternative platelet variables/tests often show little or no association, suggesting that platelet function measurements are highly assay-dependent [6,7]. These inconsistencies, combined with the wide range of available tests, make it challenging to establish the diagnostic value of platelet phenotyping. To date, no clear principles or criteria exist to differentiate patients from healthy individuals based on platelet profiles. Recent multivariate analysis (PPAnalysis) has challenged the conventional dichotomy of classifying individuals as either high- or low-responders, arguing that such stratification is overly simplistic and fails to capture biological heterogeneity. By employing exploratory statistical techniques, the investigators identified distinct subpopulations of healthy individuals, differentiated by the magnitude and pattern of their platelet responses to activating agents [8]. While this approach offers a promising alternative for understanding differences in platelet function, it remains focused on markers of platelet activation and aggregation. Notably, however, the sensitivity of platelets to platelet receptor antagonists was not assessed in this study, which seems all the stranger because it is highly relevant information in the context of potential antiplatelet therapy. The present study addresses this gap by proposing an approach that evaluates platelet responses to both agonists and inhibitors. Furthermore, this methodology can be implemented in laboratories worldwide as it relies on 96-well plate aggregometry and only requires a microplate reader—a device that is routinely available in most laboratories. High-throughput microtiter plate assays are being increasingly integrated into screening strategies and platelet phenotyping. Studies show that microtiter formats enable low-volume, parallel, multiparametric testing with high throughput, making them an excellent platform for evaluating platelet function in both human and experimental models [1,8,9,10,11].

In our study platelet phenotypes were identified through the analysis of platelet response to agonists and inhibitors of key platelet receptors, including P2Y_12_, PAR-1, and GPVI. Phenotypes were evaluated at two levels: individual receptors and globally, using multivariate analysis. This approach allowed us to examine platelet phenotypes and look into the possibility of simplifying platelet profile assessments within the population. The study provides a novel approach to assessing platelet phenotype in healthy individuals, which could potentially support clinicians in the future—once further developed—in making more personalized decisions regarding antiplatelet therapy for primary prevention of cardiovascular events.

## 2. Results

### 2.1. Characteristics of the Study Group

A panel of basic diagnostic blood tests was carried out to provide a more detailed characterisation of the study group. These included haematological variables (red blood cell count (RBC), haemoglobin (HGB), haematocrit (HCT), mean corpuscular volume (MCV), mean corpuscular haemoglobin (MCH), mean corpuscular haemoglobin concentration (MCHC), red cell distribution width (RDW), platelet count (PLT), plateletcrit (PCT), mean platelet volume (MPV), platelet distribution width (PDW), white blood cell count (WBC), neutrophil count (NEU), eosinophil count (EOS), basophil count (BAS), lymphocyte count (LYM), monocyte count (MON)) and biochemical parameters (C-reactive protein (CRP), total cholesterol (CHOL), low-density lipoprotein cholesterol (LDL), high-density lipoprotein cholesterol (HDL), triglycerides (TG)). The results are presented in Table 1. In males, the mean values of the assessed variables remained within the reference ranges. Among female participants, haematological parameters were also within normal limits. However, the biochemical profiling revealed elevated lipid values, specifically increased total cholesterol and low-density lipoprotein (LDL) concentrations.

### 2.2. Description of the Dose–Response Curves in the Platelet Aggregation Test

Dose–response curves for agonist-stimulated platelet aggregation were determined using ten different concentrations of each agonist. The dose–response curves for the inhibition of platelet aggregation were determined using nine different concentrations of each antagonist. The final ligand concentrations used are provided in Table S1. These concentrations were established through experimentation in order to achieve the widest possible range of responses (0–100%) and to accurately estimate the EC_50_/IC_50_. The following agonist concentrations were used in experiments with the inhibitors: 5 µM ADP, 3 µM TRAP-6, and 2 µg/mL CRP-A. These concentrations were empirically determined to induce a substantial platelet response. In the absence of inhibitors, the mean baseline aggregation was 56% ± 17%, 60% ± 18%, and 64% ± 17% for ADP, TRAP-6, and CRP-A, respectively.

**Figure 1:**
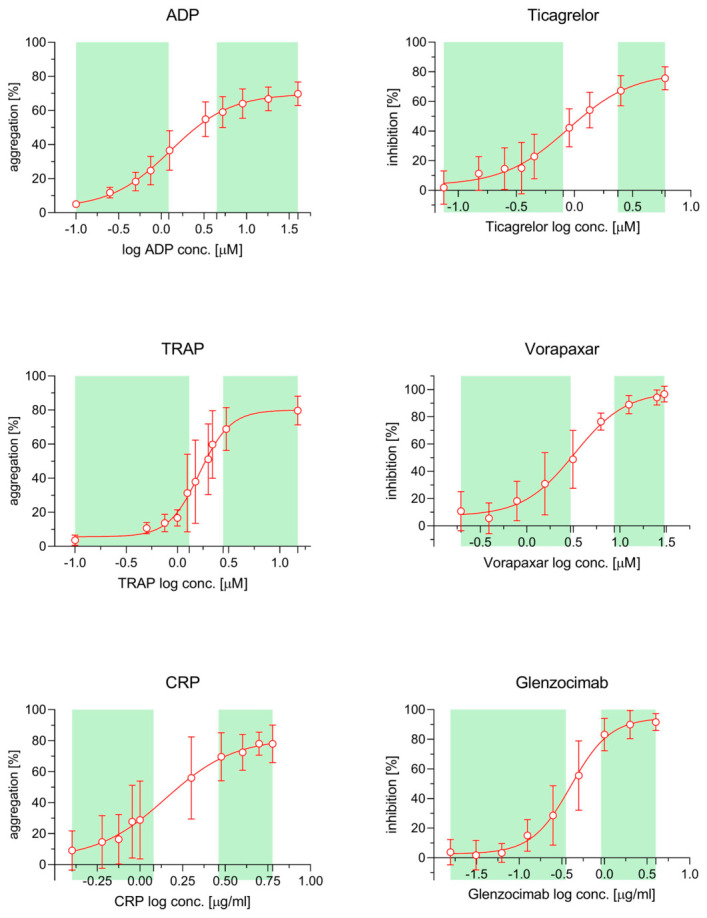
Dose–response curves of platelet aggregation in the absence and presence of selected ligands. Platelet aggregation was performed in response to ADP (0.1–40 µM), TRAP-6 (0.1–15 µM) and CRP-A (0.4–6 µg/mL). Platelet inhibitions by ticagrelor (0.075–6 µM), vorapaxar (0.2–30 µM) and glenzocimab (0.02–4 µg/mL) were measured in ADP-, TRAP-6- and CRP-A-stimulated platelets, respectively. The following concentrations were used with the inhibitors: 5 µM ADP, 3 µM TRAP-6, 2 µg/mL CRP-A. Data are shown as mean ± SE, *n* = 10. The green areas show the non-linear portions of the curves with concentration points beyond the lower and upper bend points.

Figure 1 shows the dose–response curves for platelet aggregation in response to ADP (0.1–40 µM), TRAP-6 (0.1–15 µM) and CRP-A (0.4–6 µg/mL), as well as the dose–response curves for platelet inhibition by ticagrelor (0.075–6 µM), vorapaxar (0.2–30 µM) and glenzocimab (0.02–4 µg/mL) in ADP-, TRAP-6- and CRP-A-stimulated platelets, respectively. The number of agonist and antagonist concentrations falling within the green areas (i.e., below the lower inflection point and above the upper inflection point) ranged from two to five, compared to the required minimum of two (Figure 1). The curves exhibited a characteristic sigmoidal pattern, displaying a clear plateau at higher ligand concentrations. The R^2^ values, which indicate how well the models fit the experimental data, were, on average: 0.986 for ADP, 0.972 for TRAP-6, 0.979 for CRP-A, 0.953 for ticagrelor, 0.970 for vorapaxar and 0.976 for glenzocimab. The mean EC_50_ values in the group were: 1.7 ± 1.2 µM for ADP, 1.7 ± 0.6 µM for TRAP-6, and 1.5 ± 0.7 µg/mL for CRP-A. The mean IC_50_ values were: 0.9 ± 0.5 µM for ticagrelor, 3.2 ± 1.2 µM for vorapaxar, and 0.4 ± 0.2 µg/mL for glenzocimab.

### 2.3. Distribution of Phenotype Variants

**Figure 2:**
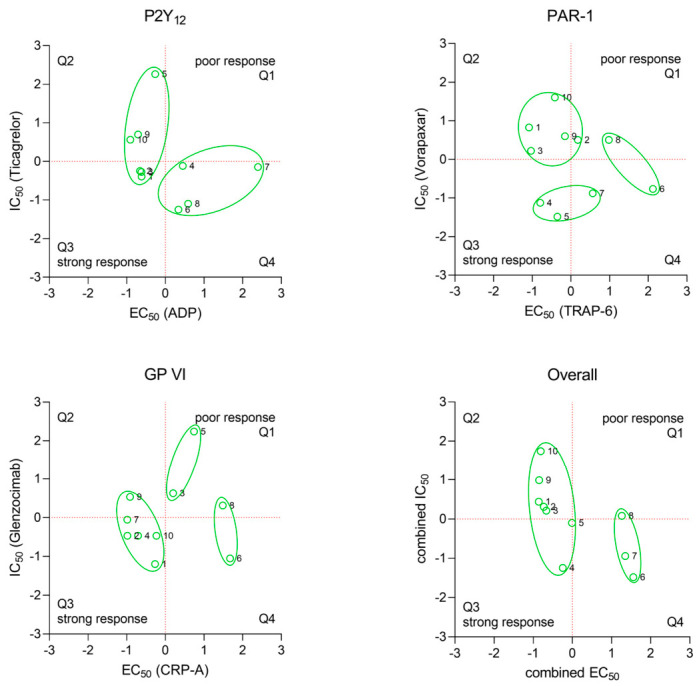
Distribution of platelet phenotypes. The platelet phenotypes of 10 healthy individuals were determined based on the EC_50_/IC_50_ values obtained for three pairs of ligands (ADP vs. ticagrelor, TRAP-6 vs. vorapaxar, and CRP-A vs. glenzocimab) for three platelet receptors: P2Y_12_, PAR-1 and GPVI. Plotting the standardized EC_50_/IC_50_ values on a coordinate plane showed that there were four possible phenotypic variants: poor platelet response (Q1), strong response (Q3) and intermediate responses (Q2 and Q4). Green circles surrounding individual data points represent clusters. For more details, please refer to Section 4.

Figure 2 illustrates the distribution of receptor-specific phenotype variants, as well as the variants dependent on the overall platelet response to agonists and antagonists. No poor responders were identified for the P2Y_12_-specific phenotype, while the proportion of other variants remained consistent at 30–40%. For the PAR-1-related phenotype, the largest subgroup (40%) consisted of individuals who were responsive to TRAP-6, but who were less sensitive to vorapaxar. The remaining variants were evenly represented, each accounting for 20%. For the GPVI-related phenotype, the largest subgroup (50%) consisted of strong responders characterized by pronounced vulnerability to CRP-A and sensitivity to glenzocimab. A considerable proportion (30%) were poor responders. The smallest subgroups (10% each) consisted of intermediate responders. Combining the results for all agonists and antagonists showed that most individuals (70%) had an intermediate response, defined as either a high EC_50_ with a low IC_50_ or vice versa. Strong and poor responders constituted the minority (Figure 2).

**Figure 3:**
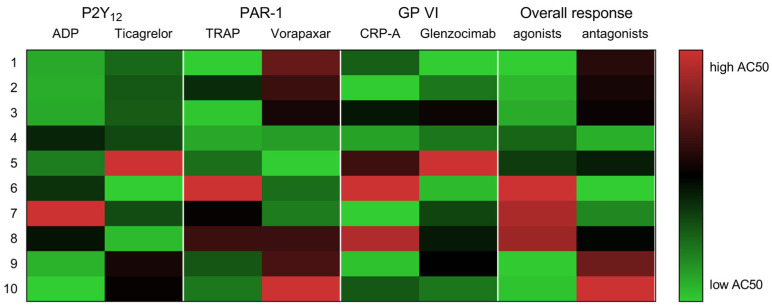
A heat map displaying an individual’s platelet responses to agonists targeting three different receptors.

Figure 3 shows a heat map summarising the results obtained from each individual in the experimental group. As can be seen, none of the individuals exhibited extreme responses (very low or very high) to both agonists and antagonists. Instead, responses varied by ligand, tending towards intermediate-to-high responses that corresponded to moderate-to-low AC_50_ values.

### 2.4. Stratification of Platelet Subsets Based on Phenotypic Similarity

First, platelet profiles were established for each participant in a two-variable analysis and then in a six-variable analysis. The two-variable clustering analysis identified two distinct clusters for the P2Y_12_-related phenotype and the overall response, as well as three clusters for the PAR-1- and GPVI-related phenotypes (Figure 2). However, conducting a six-variable clustering analysis, in which all variables are treated individually was not feasible for the experimental dataset, as this approach requires a substantially larger sample size as dimensionality increases. To overcome this limitation, we repeated the clustering procedure using a bootstrap resampling approach with replacement on the original dataset.

**Figure 4:**
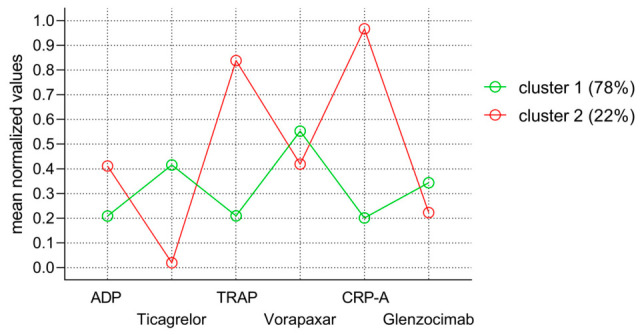
The mean normalized EC_50_ and IC_50_ values for each cluster across three different receptors in the six-variable analysis of a 100-sample bootstrap-generated dataset. The normalization procedure rescaled all AC_50_ values to the 0–1 interval, ensuring that the variables could be compared on a common scale. The k-means algorithm identified two distinct clusters.

The resulting dataset comprised 100 elements and eight clusters (Figure S1). Clusters 1 and 7 were the most stable, each containing 21 cases. The remaining clusters encompassed between five and 12 cases. In applied research, larger datasets are typically evaluated based on the assumption that clusters should contain around 20–30 objects in order to be interpretable. We therefore applied this criterion to the bootstrap-generated datasets, reducing the number of clusters to those containing a minimum of 20 cases. Accordingly, the six-variable analysis yielded two clusters (platelet variants) (Figure 4).

Based on normalised mean AC_50_ values, cluster 1 accounted for 78% of cases and comprised individuals demonstrating a strong response to agonists (low EC_50_ values for ADP, TRAP, and CRP-A) and moderate sensitivity of platelets to inhibitors (moderate IC_50_ values for all inhibitors). In contrast, cluster 2 represented 22% of cases and included individuals with a relatively low-to-moderate platelet response to agonists (high EC_50_ values for TRAP and CRP-A and moderate EC_50_ values for ADP), accompanied by moderate-to-high sensitivity to antagonists (moderate IC_50_ values for vorapaxar and low IC_50_ values for ticagrelor and glenzocimab) (Figure 4).

**Figure 5:**
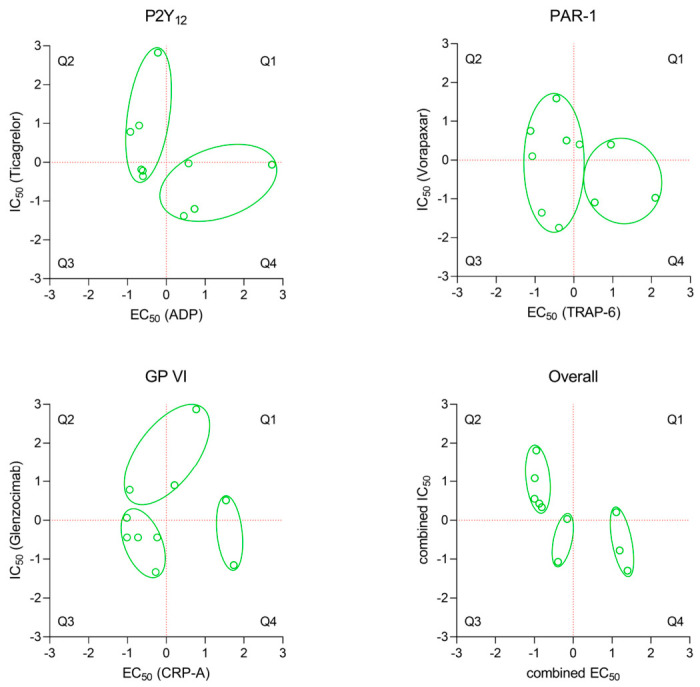
Stratification of subpopulations based on platelet responses to different ligands. Bootstrap standardised EC_50_/IC_50_ values (*n* = 100) were plotted on a coordinate plane to illustrate the distribution of poor (Q1), moderate (Q2 and Q4), and strong (Q3) responders within the cohort. The k-means method was used for clustering analysis. Two clusters were identified for P2Y_12_ and PAR-1, and three clusters for the GPVI-associated phenotype and the integrated response. The latter reflects the combined responses to all three receptor agonists and three inhibitors (two-variable analysis). The individual data points within the identified clusters were not labelled because the numerical identifiers would be illegible due to the size and spatial distribution of the datasets.

**Figure 6:**
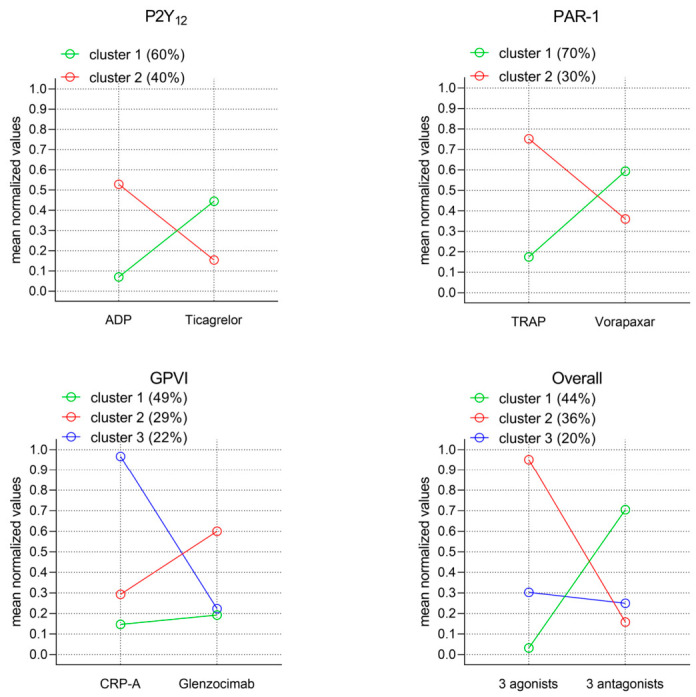
The mean normalised EC_50_ and IC_50_ values for each cluster across three receptors obtained from the two-variable analyses of bootstrap-generated datasets. Clustering was performed using the k-means algorithm.

A two-variable analysis of the bootstrap-generated dataset revealed five clusters relating to P2Y_12_- and PAR-1-related phenotypes, as well as the overall response. Furthermore, six clusters associated with the GPVI-related phenotype were identified (Figure S1). As with the six-variable analysis, the size of the clusters varied considerably, with clusters containing between five and 55 cases. Reducing the number of clusters to those containing a minimum of 20 cases produced two clusters relating to the P2Y_12_- and PAR-1-related phenotypes, as well as three clusters relating to the GPVI-related phenotype and the overall response (Figure 5). Figure 6 shows the detailed characteristics of the observed clusters alongside the proportion of cases in each cluster.

### 2.5. Relationship Between Platelet Responses to Agonists and Sensitivity to Inhibitors

We also examined the relationships between platelet responses to receptor-specific ligands such as ADP vs. ticagrelor, TRAP 6 vs. vorapaxar and CRP A vs. glenzocimab in a bootstrap-generated dataset. We found that the aggregatory response of platelets to P2Y_12_ and PAR-1 agonists was inversely proportional to that of their respective inhibitors. In contrast, the platelet response to CRP-A for GPVI was directly proportional to the response to glenzocimab. However, these relationships were only significant for P2Y_12_ (R_S_ = −0.359, *p* < 0.0001) and PAR-1 (R_S_ = −0.214, *p* < 0.033), but not for GPVI (R_S_ = 0.079, *p* = 0.432).

## 3. Discussion

This article presents a new method of platelet phenotyping that combines responses to agonists and inhibitors of platelets using exploratory analytical techniques. A comprehensive analysis of signalling pathways via the P2Y_12_, PAR-1 and GPVI receptors was conducted in order to accurately characterise platelet reactivity and sensitivity. Incorporating inhibitors into platelet phenotype profiling provides valuable insights that may, in the future and with further validation, contribute to informing antiplatelet therapy in clinical practice. Furthermore, the selected methodology is distinguished by its high degree of flexibility and considerable potential. Consequently, this approach can be used to identify platelet phenotypes associated with any selected signalling pathway. The necessary analytical tools are inexpensive and widely available, making the methodology suitable for research and clinical settings.

### 3.1. Platelet Phenotypes Identified Among Healthy Individuals

**Figure 7:**
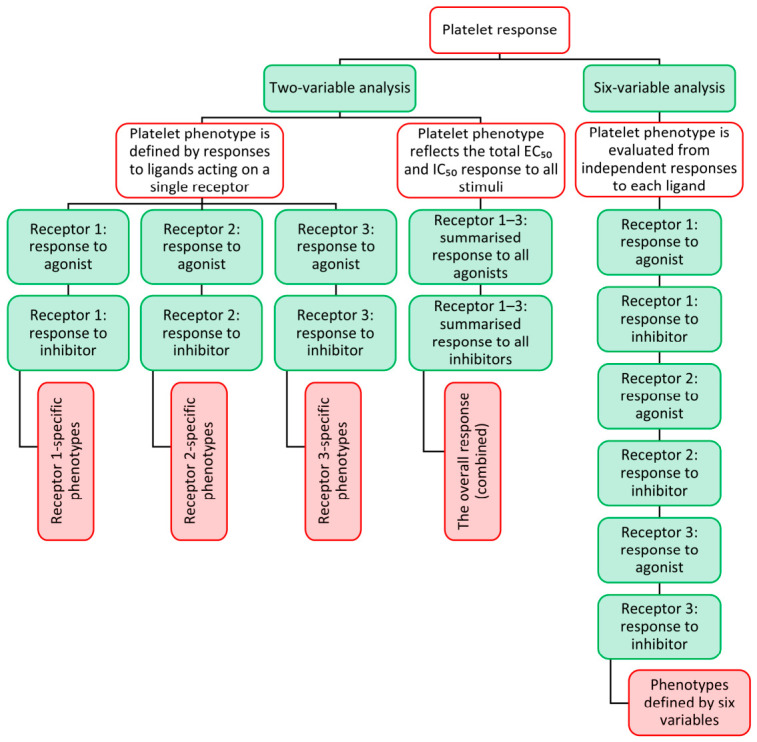
Study design. The following flowchart illustrates a strategy for profiling platelet phenotypes. This incorporates two- and six-variable analyses of the platelet response to targeted receptor stimulation (three receptors: P2Y_12_, PAR-1 and GPVI), comparing the effects of agonists and antagonists. In the two-variable analysis, a platelet phenotype is defined as the response to two ligands (one agonist and one antagonist) acting on one receptor. Alternatively, it is defined as the summed EC_50_ and IC_50_ responses to all agonists and inhibitors (i.e., the overall response). In the six-variable analysis, platelet phenotypes are classified as sets of independent responses to different modulators. This dual-level approach enables high-throughput characterisation of platelet function and inter-individual variability in pharmacological responsiveness.

Platelet phenotypes were identified based on responses mediated through three major receptors—P2Y_12_, PAR-1 and GPVI—using their respective agonists (ADP, TRAP-6 and CRP-A) and inhibitors (ticagrelor, vorapaxar and glenzocimab). The resulting phenotypes could therefore be defined in relation to these receptor-specific signalling pathways. Phenotypic variants were profiled through the implementation of two- and six-variable cluster analyses (Figure 7).

However, the implementation of the latter was only made possible with the bootstrapped datasets generated from the experimental data (see below for limitations). Consequently, the interpretation provided below is restricted to these larger datasets. The two- and six-variable analyses revealed two primary platelet variants within the datasets (Figure 4 and Figure 6). Variant 1 comprised individuals with a relatively high response to agonists (low EC_50_ values) and relatively moderate to low sensitivity to inhibitors (moderate to high IC_50_ values). Variant 2 comprised cases exhibiting a moderate to low response to agonists (moderate to high EC_50_ values) and moderate to high sensitivity to inhibitors (moderate to low IC_50_ values). Furthermore, a substantial proportion of cases exhibited characteristics typical of variant 1 in the two-variable analysis: 60% for P2Y_12_, 70% for PAR-1 and 44% for the overall response (Figure 6, cluster 1) and 29% for GPVI (Figure 6, cluster 2). In the six-variable analysis 78% of cases showed features consistent with variant 1 (Figure 4, cluster 1). An additional variant was identified in the analysis of the collagen receptor (GPVI)-related phenotype, accounting for up to 49% of cases (Figure 6, cluster 1). This cluster included individuals exhibiting a high response to GPVI agonist and antagonist (low EC_50_ and IC_50_ values). These findings most likely influenced the results obtained for the overall platelet response, for which all three of the aforementioned platelet variants (Figure 6, clusters 1–3) were observed. However, the frequency of these variants differed from that observed for GPVI. As shown in Figure 6, in the overall response, variant 1 (cluster 1) was dominant, while variant 3 (cluster 3) occurred with a lower frequency of 20%.

It is noteworthy that the number of clusters and thus the number of platelet variants given above is considerably lower than in the PPAnalysis study. In the latter study, a human cohort was divided into six subgroups using multiparametric hierarchical clustering [8]. This discrepancy can be attributed to the methodological differences between the two studies. A fundamental distinction was the stipulation that each cluster should comprise a minimum of 20 objects. Taking this constraint into account, the maximum number of platelet variants that can theoretically be postulated within the dataset under scrutiny is five. Initial elbow-method calculations indicated five to six clusters in the two-variable analysis and eight clusters in the six-variable analysis (Figure S1). However, a considerable proportion of the clusters did not meet the minimum size threshold. Consequently, the number of clusters was reduced to two or three in order to satisfy this criterion. While this approach facilitates the interpretation of the data, it inevitably results in a loss of information. For example, prior to cluster reduction, a group of high responders (platelet variant 3) was identified for PAR-1 (Figure S1, cluster 1). Furthermore, a small cluster characterised by a moderate response to CRP-A (moderate EC_50_) and relatively low sensitivity to glenzocimab (high IC_50_) was identified (Figure S1, cluster 2).

These findings emphasise the importance of ensuring an adequately large sample size in order to maximise the amount of information that can be obtained from such analyses. Notably, no low responders (high EC_50_ and high IC_50_ values) were identified in the present study. This may be because our findings are based on experimental data collected from healthy individuals. In clinical practice, however, individuals with hyperreactive platelets require focused attention, particularly those with low EC_50_ and high IC_50_ values. Hyperreactive platelets, characterised by an enhanced response to agonists, are recognised as a risk factor for atherosclerosis and major cardiovascular events [12]. In clinical settings, this is often referred to as residual platelet activity following antiplatelet therapy. Persistent hyperreactivity of this kind has been shown to be associated with adverse cardiovascular outcomes in patients with acute coronary syndromes [13]. Consequently, categorising platelets using the proposed methodology could help to quickly identify individuals at high risk of cardiovascular disease. This would allow personalised antiplatelet therapy to be provided to those who need it.

### 3.2. Methodological Aspects

In the present study, the platelet aggregation assay was used for platelet phenotyping, a well-established method. Yee et al. adopted conventional light transmission aggregometry (LTA) to identify a hyperreactive platelet phenotype in vitro among 359 healthy individuals [3,14]. They demonstrated that submaximal concentrations of epinephrine can effectively distinguish individuals with hyperreactive platelets. Furthermore, they showed that this phenotype is consistent and enduring, with high temporal reproducibility and persistence for up to three years. Evidence from various methodological approaches also indicates that platelet hyperreactivity extends beyond epinephrine-mediated aggregation to include multiple facets of platelet function, such as increased adhesion, activation and aggregation in response to shear stress and various other agonists. Similarly, Berger et al. investigated the biological basis of platelet hyperreactivity using LTA to demonstrate the influence of critical factors such as serotonin kinetics and transporter function on this phenotype [15]. The Platelet Reactivity Expression Score (PRESS) is a recently developed, innovative tool capable of identifying individuals with platelet hyperreactivity [16]. A notable feature of the study is the integration of platelet aggregation responses with RNA sequencing. Using the PRESS methodology, researchers have demonstrated a link between increased platelet reactivity in patients with peripheral artery disease (PAD) and a higher risk of major cardiovascular events within 30 days of revascularisation—almost three times higher than expected. The score has also been shown to effectively predict cardiovascular risk in healthy individuals. Although platelet transcriptome analysis is a highly promising approach for diagnosis, prognosis assessment, and therapy monitoring—including in oncology—its clinical application is currently limited by technical constraints, high costs, and insufficient validation [17].

It is acknowledged that the conventional light transmission assay is a low-throughput test. Therefore, the present study employed microplate-based aggregation to enable higher-throughput analysis. The difference between the two types of assay is that microtiter plate-based platelet aggregation measures light absorbance rather than light transmission [18,19]. A comprehensive review of 96-well plate aggregometry was recently published [20]. As previously described [18,19,20,21,22,23,24,25], this method offers numerous advantages, facilitating the implementation of our methodology using specific ligand combinations. Although microplates can be pre-coated to streamline the workflow [19,22], all reagents in this study were freshly prepared prior to use. Several studies have demonstrated the potential of a microtiter plate-based platelet aggregation assay as a valuable tool for screening bleeding disorders and monitoring antiplatelet therapy, showing a high correlation with LTA [21,24]. Because it can detect both mild and severe platelet function disorders, the method has recently been used to validate the new flow cytometry-based PPAnalysis platform [8].

However, despite these advantages, 96-well platelet aggregometry, like other platelet function tests, remains poorly standardised, as implementing routine quality-control procedures in platelet studies continues to pose a significant challenge. Key variables that can introduce artefacts into platelet analysis include the choice and concentration of agonists, as well as the protocols used for blood collection and sample processing [26]. To minimise the risk of such artefacts, the experimental procedures used in this study adhered to established guidelines designed to prevent premature platelet activation and aggregation (see Section 4 for details). All experiments were also performed within four hours of collection and used a fixed, standardised platelet count, since results are sensitive to both the time elapsed since collection and the final cell number.

Alongside the aggregation tests, basic diagnostic assessments were performed on the donors. These revealed that women had significantly higher cholesterol levels than men. However, it is hypothesised that this difference did not significantly influence the aggregation measurements. Blood was collected in a fasting state, and no adverse responses to ligands were observed. Additionally, the plasma samples used for the measurements were not lipemic—notably, the baseline plasma absorbance ranged from 0.048 to 0.061, and did not differ by sex. Finally, as noted in the LTA guidelines, the extent to which hyperlipidaemia hinders the diagnosis of platelet function remains unclear [27].

The presence of procoagulant platelets that are unable to aggregate could potentially affect the outcome of platelet aggregation tests. However, previous studies that have provided detailed insights into the kinetics and determinants of procoagulant platelet formation suggest that their contribution to the observed platelet response was likely negligible under the conditions applied in our study, if it existed at all. It is well established that, prior to agonist stimulation, platelets constitute a single, homogeneous population of relatively uniform size. This population is characterised by a near-complete absence of phosphatidylserine (PS) exposure and a preserved mitochondrial membrane potential. Upon stimulation with strong agonists, however, this population loses its homogeneity and segregates into distinct subpopulations, including a subset of platelets with a procoagulant phenotype [28,29]. Crucially, the generation of procoagulant platelets requires potent stimuli that can induce a sustained increase in cytosolic calcium exceeding a defined threshold. The procoagulant phenotype is typically activated using a combination of potent agonists, most commonly thrombin and collagen, because agonists such as ADP or thrombin alone have little or no effect. Furthermore, the formation of a stable procoagulant platelet subpopulation generally requires 10–30 min [28,29,30,31,32]. In contrast, our experimental protocol employed a 5 min stimulation with a single agonist (ADP, TRAP-6, or CRP-A). Under these conditions, it is unlikely that a significant proportion of procoagulant platelets would be produced and affect aggregation. This conclusion is further supported by the consistency of the EC_50_ values obtained for the agonists used and the published data. Nevertheless, future studies could benefit from quantifying procoagulant platelets after measuring platelet aggregation, particularly following co-stimulation with a GPVI agonist and thrombin. This is because signalling through the GPVI receptor is a key pathway in procoagulant platelet formation. It is worth noting that the quantification of procoagulant platelets is not included in current LTA recommendations. Consequently, they are not included in standard aggregation measurements.

### 3.3. Limitations

This study uses a methodology that makes it possible to analyse phenotypes defined by responses to ligands that act on platelet receptors. While the experimental data allowed for the presentation of a novel methodology, the primary limitation of the study was the small sample size, which prevented multi-variable analysis. Another issue relating to the sample size concerns the clustering strategy of the bootstrapped data, which relied on an arbitrary threshold that could mask meaningful subgroups. This was done at an analytical level to identify stable clusters of moderate to large size. Bootstrap resampling was applied to compensate for the small sample size. However, this approach only captures the variability inherent in the existing experimental data. With a larger sample size, biological variability would likely increase, potentially revealing additional phenotypes, such as low responders. Similarly, the use of ligands other than those used in the study might influence the platelet phenotypic map, as different ligands could induce distinct signalling dynamics and shape the platelet response differently. Nevertheless, bootstrap resampling enabled us to demonstrate the relationship between platelet responses to ligands and suggest that dimensionality re-duction using principal component analysis (PCA) could be a viable option when working with larger experimental datasets. Our data showing associations between agonists and antagonists of the same receptor are consistent with the findings of Carazo et al., who recently reported correlations among platelet aggregation pathways in a cohort of healthy volunteers [33]. These studies showed that responses to one inducer could predict responses to other triggers or antiplatelet drugs. Overall, this study introduces a new methodology and establishes a foundation for the large-scale stratification of healthy individuals. However, the results should be interpreted with caution, as the analysis is based on a small group.

## 4. Materials and Methods

### 4.1. Chemicals

Adenosine 5′-diphosphate (ADP) sodium salt, dimethyl sulfoxide (DMSO), and gelatin were obtained from Sigma (St. Louis, MO, USA). Thrombin receptor agonist peptide (TRAP-6 amide) was purchased from Bachem AG (Bubendorf, Switzerland). Collagen-related peptide (CRP-A) was purchased from Pplus Skin Care Limited (Salisbury, UK). Ticagrelor was purchased from Tocris Bioscience (Bristol, UK). Vorapaxar was from Axon Medchem B.V. (Groningen, The Netherlands). Glenzocimab (anti-GPVI mAb; RRID: AB 3695364) was purchased from MedChemExpress LLC (Monmouth Junction, NJ, USA). Dulbecco’s Phosphate-Buffered Saline (DPBS) was obtained from Mediatech Inc. (Manassas, VA, USA). Sodium chloride solution (0.9%) was from Glenmark Pharmaceuticals (Warsaw, Poland). Blood collection tubes containing a buffered sodium citrate solution (0.109 M) were obtained from Becton, Dickinson and Company (San Diego, CA, USA). S-Monovette^®^ tubes and needles were from Sarstedt (Numbrecht, Germany). Ninety-six well polystyrene (PS), half-area, flat-bottom ELISA plates, and 96-well polypropylene (PP) flat-bottom ELISA plates were from Greiner Bio-One (Frickenhausen, Germany).

### 4.2. Study Design

The characterization of platelet phenotypes in healthy individuals was based on the analysis of platelet responses to agonists and inhibitors of a few selected key receptors, including P2Y_12_, PAR-1, and GPVI. Specifically, experiments targeted three receptor systems, each examined with a pair of ligands (one agonist and one antagonist): (1) ADP with ticagrelor; (2) TRAP with vorapaxar; and (3) CRP-A with glenzocimab. The experimental design required an assay capable of quantifying platelet function rapidly and efficiently. To this end, light transmission aggregometry (LTA) was employed in a microplate format—specifically, a 96-well half-area plate—to perform measurements in platelet-rich plasma (PRP).

This method successfully determined the half-maximal effective concentrations (EC_50_) of the platelet agonists (ADP, TRAP and CRP-A) and the potencies of the platelet inhibitors (ticagrelor, vorapaxar and glenzocimab) (IC_50_) in samples from each individual. The effects of the inhibitors on platelet function were assessed following stimulation with ADP, TRAP and CRP-A, respectively. Platelet phenotypes in the group were profiled using two- and six-variable analyses. Figure 7 shows a schematic diagram of the study design.

### 4.3. Preparation of Compound Solutions

Stock solutions of the agonists (ADP, TRAP and CRP-A) and glenzocimab were prepared in sterile saline. Stock solutions of ticagrelor and vorapaxar were prepared in DMSO. All stock solutions were stored at −80 °C until use. Working solutions of the examined ligands were freshly prepared in the same solvents as the stock solutions by diluting them to concentrations being 10, 50 or 300 times higher than the intended final concentrations, depending on the model (stimulation or inhibition of agonist-stimulated cells) and the solubility of the ligands. The final concentration of dimethyl sulfoxide (DMSO) in the tested samples was 0.3%, regardless of the inhibitor concentration (ticagrelor or vorapaxar). This concentration did not affect platelet aggregation.

### 4.4. Participants

Human blood was collected from ten healthy volunteers: 4 males and 6 females with a mean age of 47 ± 10 years. All individuals gave their written informed consent to participate in the study. All subjects confirmed that they had not taken any medication known to affect platelet function, such as non-steroidal anti-inflammatory drugs (NSAIDs), for at least two weeks prior to the study. The study was approved by the Ethics Committee of the Medical University of Łódź, Poland (RNN/130/23/KE), and was conducted in accordance with the Declaration of Helsinki.

### 4.5. Blood Collection and Processing

Blood samples were taken from fasting participants between 8.00 and 9.00 a.m., using light tourniquets and short, moderate phlebostasis, from the antecubital vein with 19-gauge needles. The first few millilitres of blood were collected in EDTA and serum tubes to perform a complete blood count (CBC), a lipid profile test and a C-reactive protein test. Blood samples for platelet testing were collected in accordance with ISTH recommendations for the standardisation of light transmission aggregometry [27] and were consistent with the guidelines for the laboratory investigation of heritable disorders of platelet function [34]. Samples were collected in polypropylene S-Monovette^®^ tubes prefilled with a 0.109 M sodium citrate solution at a blood/anticoagulant ratio of 9:1 (vol/vol). The tubes were gently inverted several times immediately after blood collection to ensure proper mixing with the anticoagulant. Platelet-rich plasma (PRP) was prepared by centrifuging the collected blood at 200× *g* for 12 min at 37 °C using a Jouan MR1822 centrifuge (Saint-Herblain, France). Platelet-poor plasma (PPP) was obtained by centrifuging the PRP samples at 2000× *g* for 20 min at 37 °C using a Sigma 2-16PK centrifuge (Osterode, Germany). The platelet count in the PRP was measured using an Sysmex XS-800i automated haematology analyser (Sysmex, Kobe, Japan).

### 4.6. Ninety-Six Well Aggregometry

Platelet aggregation was quantified at a cell count of 3 × 10^8^ cells per ml in 96-well polymer microtiter plates that had been pre-coated with 0.75% gelatin overnight at 4 °C to prevent platelet activation. The platelet response to the agonists (ADP, TRAP-6, and CRP-A), both with and without the inhibitors (ticagrelor, vorapaxar, and glenzocimab), was examined in a final volume of 50 µL, as previously described [35]. Briefly, the microplate wells were filled with 5 µL of agonist and 45 µL of sample (PRP). The microtiter plates were then sealed with a sealing film and shaken at 1200 rpm for five minutes at 37 °C using a Titramax 100 shaker (Heidolph Scientific Products GmbH, Schwabach, Germany) to promote platelet aggregation. Immediately after shaking, the absorbance was measured at 595 nm using a VICTOR™ X4 microplate reader (PerkinElmer, Waltham, MA, USA). The PRP samples were pre-incubated with the inhibitors at 37 °C for 10 min without shaking (samples were gently mixed to prevent premature platelet activation). They were then transferred to wells containing either ADP, TRAP-6 or CRP-A, depending on whether the cells had been treated with ticagrelor, vorapaxar or glenzocimab. Control wells contained saline instead of an agonist. PPP was used instead of PRP for the blank sample. All experiments were carried out within four hours of blood collection.

### 4.7. Statistical Analysis

The results are presented as the arithmetic mean ± SD. EC_50_ and IC_50_ values were determined using four-parameter logistic (4PL) non-linear regression analysis of dose–response curves. The methodology developed by Sebaugh [36] was employed to accurately estimate these values.

The platelet profile of each participant was established using either two or six variables. In the two-variable analysis, receptor-specific phenotypes and the overall response were evaluated. The six-variable analysis classified phenotypic variants based on similarities and differences in the EC_50_/IC_50_ values of individual ligands, reflecting the platelet response to all six ligands (Figure 7). To assess receptor-specific phenotypes, the EC_50_/IC_50_ values obtained for each ligand pair were standardised (value − mean)/standard deviation) and plotted on a coordinate plane. To evaluate the overall response, the EC_50_/IC_50_ values for all agonists and antagonists were summed, standardised and plotted on a coordinate plane. The two-variable analysis revealed the distribution of four distinct phenotypic variants of platelet responses to the drugs: poor response (high EC_50_/high IC_50_, top right quadrant, Q1), strong response (low EC_50_/low IC_50_, bottom left quadrant, Q3), and intermediate response (low EC_50_/high IC_50_, top left quadrant, Q2 or high EC_50_/low IC_50_, the bottom right quadrant, Q4). To facilitate comprehensive visualisation, heat maps were generated for each receptor individually and after integration (by summing the EC_50_ and IC_50_ values). The AC_50_ measure was used to compare the activity of agonists and inhibitors. This is defined as the concentration of a substance at which 50% of maximal biological activity is achieved in the assay.

Two- and six-variable cluster analyses were performed using the k-means algorithm. As this method requires larger datasets as dimensionality increases, a bootstrap resampling approach (100 iterations) was applied to the sample consisting of 10 sets (10 individuals) of three pairs of experimental data points (three pairs of agonist–inhibitor). V-fold cross-validation and the elbow method were subsequently used to determine the optimal number of clusters. Spearman’s rank correlation was used to evaluate correlations between variables. Statistical analyses were conducted using Statistica v. 13.3, GraphPad Prism v. 8.0.1 and Resampling Stats Add-in for Excel v. 4.0.

## 5. Conclusions

This study makes a valuable contribution to the ongoing discussion on multiparameter platelet phenotyping analysis by proposing a novel methodology for evaluating platelet phenotypes in both healthy individuals and patients who are not receiving antiplatelet therapy. This strategy enables a more detailed characterisation of platelet responses to agonists and antagonists, which could help to identify individuals who are predisposed to cardiovascular events. Following further validation, the proposed method could also support more personalised decision-making in antiplatelet therapy for the primary prevention of such events. Within the context of emerging multi-omic approaches to platelet heterogeneity that integrate progenitor, megakaryocyte, and platelet-level analyses from the same patient [4], our platelet phenotyping framework could be a key component. By providing functional information at the platelet level, this methodology can complement transcriptomic and other omic datasets, thereby supporting a more comprehensive multi-layered analysis of platelet heterogeneity and its biological and clinical implications.

## Figures and Tables

**Table 1:** Selected haematological and biochemical variables measured in the examined individuals.

Variable	Males	Reference Range	Females	Reference Range
*n*	4		6	
age [years]	50 ± 13		45 ± 7	
RBC [×10^12^/L]	4.95 ± 0.28	4.20–6.10	4.42 ± 0.28	3.70–5.10
HGB [g/dL]	15.2 ± 0.8	14.0–18.0	12.7 ± 0.9	12.0–16.0
HCT [%]	44.3 ± 1.7	40.0–55.0	38.1 ± 2.2	36.0–48.0
MCV [fL]	90 ± 5	80–98	87 ± 6	80–98
MCH [pg]	30.7 ± 1.3	26.0–34.0	28.7 ± 2.4	26.0–34.0
MCHC [g/dL]	34.3 ± 0.7	31.0–36.0	33.3 ± 0.8	31.0–36.0
RDW [%]	12.5 ± 0.4	11.0–15.5	13.1 ± 0.7	11.0–15.5
PLT [×10^9^/L]	248 ± 58	150–400	265 ± 77	150–400
PCT [%]	0.25 ± 0.04	0.20–0.50	0.30 ± 0.06	0.20–0.50
MPV [fL]	9.9 ± 0.6	8.0–12.0	11.4 ± 1.2	8.0–12.0
PDW [fL]	11.1 ± 1.3	9.0–17.0	14.4 ± 3.4	9.0–17.0
WBC [×10^6^/L]	4.83 ± 0.87	4.00–11.00	5.34 ± 1.20	4.00–11.00
NEU [×10^6^/L]	2.71 ± 0.56	2.20–4.80	3.11 ± 1.16	2.20–4.80
EOS [×10^6^/L]	0.10 ± 0.04	0.00–0.20	0.19 ± 0.10	0.00–0.20
BAS [×10^6^/L]	0.04 ± 0.02	0.00–0.10	0.05 ± 0.02	0.00–0.10
LYM [×10^6^/L]	1.49 ± 0.30	1.30–2.90	1.53 ± 0.44	1.30–2.90
MON [×10^6^/L]	0.48 ± 0.05	0.30–0.80	0.44 ± 0.09	0.30–0.80
CRP [mg/L]	0.8 ± 0.5	0.0–5.0	2.5 ± 3.6	0.0–5.0
CHOL [mmol/L]	4.57 ± 0.63	3.00–5.00	5.80 ± 0.82	3.00–5.00
LDL [mmol/L]	2.94 ± 0.53	<3.00	3.50 ± 0.67	<3.00
HDL [mmol/L]	1.44 ± 0.09	>1.00	1.81 ± 0.21	>1.20
TG [mmol/L]	0.91 ± 0.16	<1.70	1.12 ± 0.49	<1.70

Data are shown as mean ± SD. The reference ranges intervals for haematological and biochemical blood parameters were established by the local laboratory. Furthermore, women had significantly higher total cholesterol levels than men (*p* < 0.05).

## Data Availability

The data that support the findings of this study are available from the corresponding author upon reasonable request. Some data may not be made available because of privacy or ethical restrictions.

## Supplementary Materials

The following supporting information can be downloaded at: https://www.mdpi.com/article/10.3390/ph19060821/s1, Figure S1: The mean normalised EC_50_ and IC_50_ values calculated for each cluster in two- and six-variable analyses of the 100-sample bootstrap-generated datasets; Table S1: The final concentrations of platelet agonists and inhibitors used in the study.

## Abbreviations

The following abbreviations are used in this manuscript:

CRP	C-reactive protein
CRP-A	Collagen-related peptide
GPVI	Glycoprotein VI
LTA	Light transmission aggregometry
PPP	Platelet-poor plasma
PRP	Platelet-rich plasma
