# Supplementary material for: A Practical Approach to Platelet Phenotype Profiling Using Microplate Aggregometry
Source: Pharmaceuticals (Basel). 2026 May 23;19(6):821. doi: 10.3390/ph19060821 (PMC13306059; doi:10.3390/ph19060821)
Supplement: Supplementary file 1 [file pharmaceuticals-19-00821-s001.zip › pharmaceuticals-4238987-supplementary.pdf]

**A practical approach to platelet phenotype profiling using microplate aggregometry**

Supplementary material

**Table S1.** The final concentrations of platelet agonists and inhibitors used in the study.

| ADP [µM] | Ticagrelor [µM] | TRAP-6 [µM] | Vorapaxar [µM] | CRP-A [µg/ml] | Glenzocimab [µg/ml] |
|---|---|---|---|---|---|
| 40.00 | 6.00 | 15.00 | 30.00 | 6.00 | 4.00 |
| 18.00 | 2.50 | 3.00 | 25.00 | 5.00 | 2.00 |
| 9.00 | 1.35 | 2.20 | 12.50 | 4.00 | 1.00 |
| 5.25 | 0.90 | 2.00 | 6.25 | 3.00 | 0.50 |
| 3.30 | 0.45 | 1.50 | 3.12 | 2.00 | 0.25 |
| 1.25 | 0.35 | 1.25 | 1.56 | 1.00 | 0.12 |
| 0.75 | 0.25 | 1.00 | 0.78 | 0.90 | 0.06 |
| 0.50 | 0.15 | 0.75 | 0.39 | 0.75 | 0.03 |
| 0.25 | 0.07 | 0.50 | 0.20 | 0.60 | 0.02 |
| 0.10 | | 0.10 | | 0.40 | |

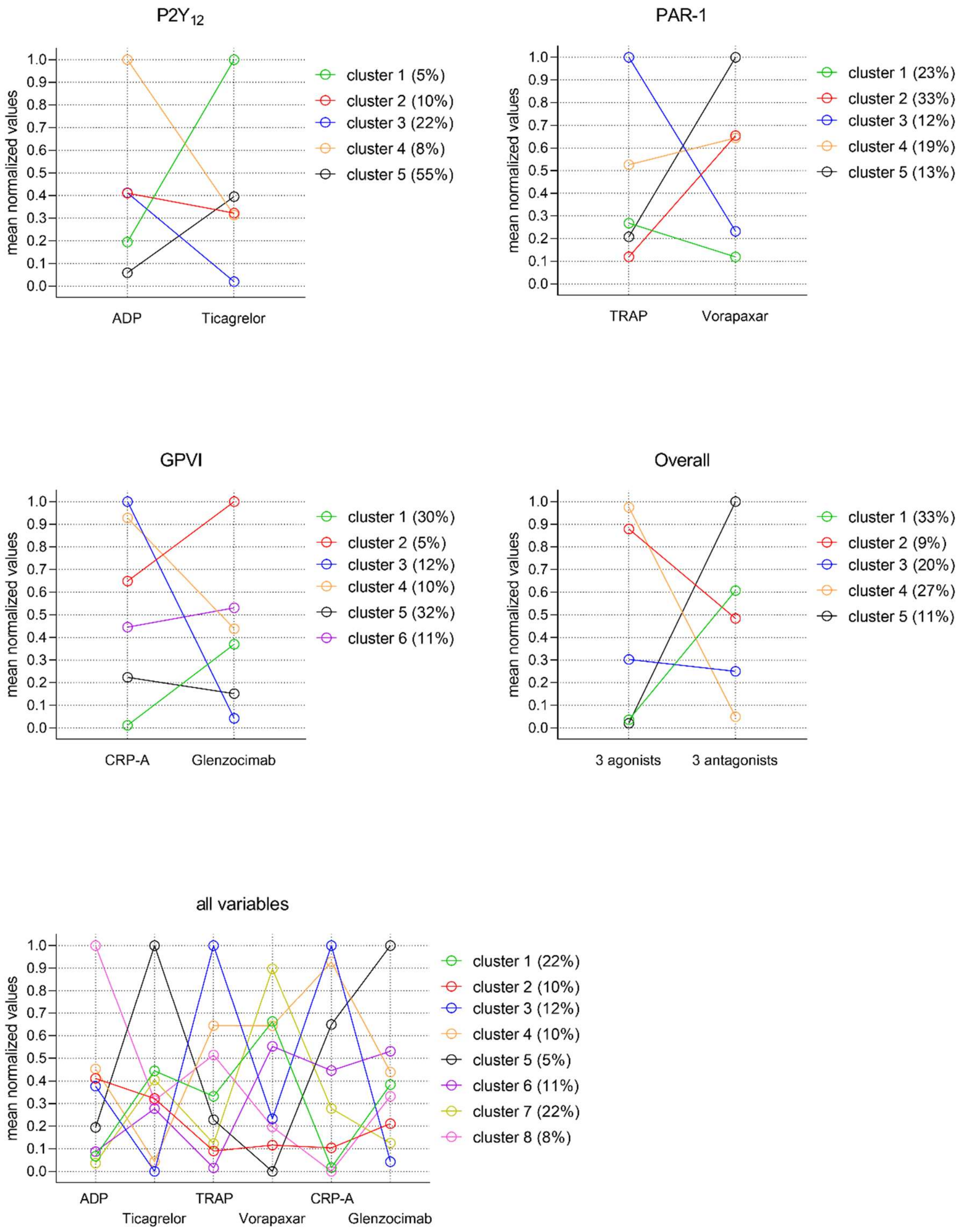


**Figure S1.** The mean normalised $EC_{50}$ and $IC_{50}$ values calculated for each cluster in two- and six-variable analyses of the 100-sample bootstrap-generated datasets. The elbow method was used to determine the optimal number of clusters for the k-means algorithm.
